# Genetic diversity and structure in hill rice (*Oryza sativa* L.) landraces from the North-Eastern Himalayas of India

**DOI:** 10.1186/s12863-016-0414-1

**Published:** 2016-07-13

**Authors:** Somnath Roy, B. C. Marndi, B. Mawkhlieng, A. Banerjee, R. M. Yadav, A. K. Misra, K. C. Bansal

**Affiliations:** ICAR-National Bureau of Plant Genetic Resources, Regional Station, Umiam, Meghalaya 793103 India; ICAR-National Rice Research Institute, Cuttack, Odisha 753006 India; ICAR Research Complex for NEH Region, Umiam, Meghalaya 793103 India; ICAR-National Bureau of Plant Genetic Resources, New Delhi, 110012 India; Present address: ICAR-National Rice Research Institute, Cuttack, Odisha 753006 India

**Keywords:** Rice (*Oryza sativa*), Diversity, Landrace, Hill rice, Exploration, Northeast India

## Abstract

**Background:**

Hill rices (*Oryza sativa* L.) are direct seeded rices grown on hill slopes of different gradients. These landraces have evolved under rainfed and harsh environmental conditions and may possess genes governing adaptation traits such as tolerance to cold and moisture stress. In this study, 64 hill rice landraces were collected from the state of Arunachal Pradesh of North-Eastern region of India, and assessed by agro-morphological variability and microsatellite markers polymorphism. Our aim was to use phenotypic and genetic diversity data to understand the basis of farmers’ classification of hill rice landraces into two groups: *umte* and *tening*. Another goal was to understand the genetic differentiation of hill rices into *Indica* or *japonica* subspecies.

**Results:**

According to farmers’ classification, hill rices were categorized into two groups: *umte* (large-grained, late maturing) and *tening* (small-grained, early maturing). We did not find significant difference in days to 50 % flowering between the groups. Principal component analysis revealed that two groups can be distinguished on the basis of kernel length-to-width ration (KLW), kernel length (KL), grain length (GrL), grain length-to-width ration (GrLW) and plant height (Ht). Stepwise canonical discriminant analysis identified KL and Ht as the main discriminatory characters between the cultivar groups. Genetic diversity analysis with 35 SSR markers revealed considerable genetic diversity in the hill rice germplasm (gene diversity: 0.66; polymorphism information content: 0.62). Pair-wise allelic difference between *umte* and *tening* groups was not statistically significant. The model-based population structure analysis showed that the hill rices were clustered into two broad groups corresponding to *Indica* and *Japonica*. The geographic distribution and cultivars grouping of hill rices were not congruent in genetic clusters. Both distance- and model-based approaches indicated that the hill rices were predominantly *japonica* or admixture among the groups within the subspecies. These findings were further supported by combined analysis hill rices with 150 reference rice accessions representing major genetic groups of rice.

**Conclusion:**

This study collected a valuable set of hill rice germplasm for rice breeding and for evolutionary studies. It also generated a new set of information on genetic and phenotypic diversity of hill rice landraces in North-Eastern region of India. The collected hill rices were mostly *japonica* or admixture among the subpopulations of *Indica* or *Japonica.* The findings are useful for utilization and conservation of hill rice germplasm.

**Electronic supplementary material:**

The online version of this article (doi:10.1186/s12863-016-0414-1) contains supplementary material, which is available to authorized users.

## Background

Rice is the primary food source for more than half of the world’s population [1]. Over 90 % of the world’s rice is produced and consumed in the Asia-Pacific region. Asian cultivated rice (*Oryza sativa* L.) is one of the important crops in the world. Being a model organism with fully sequenced genome, it also affords unique opportunities to use genomic approaches to study its domestication, adaptive selection, and the history of crop improvement [2, 3]. The genetic structure of rice on a global scale is well characterized (see reviews in [4–6]). In addition to the two widely accepted major subspecies, *Indica* and *Japonica*, three to seven genetically distinct groups have been identified within global rice (*Oryza sativa*) germplasm sets in different studies [7–14]. Numerous studies on genetic structure of rice cultivars at a local scale (within a country) have also been conducted [15–22]. Such local-scale studies not only provide a detail view of rice genetic diversity within a country, but also allow for a better understanding of complex interaction between rice genetic diversity and human cultivation practices [18], and for formulating in situ conservation strategies [17]. The cultivated rice genetic diversity includes indigenous landraces and commercially bred improved varieties. As a consequence of both natural and artificial selection under diverse habitats, high levels of morphological, physiological and genetic diversity exist within *O. sativa*, resulting in >120,000 distinct rice varieties [1, 23]. An assessment of this diversity is important for making decision about the conservation and effective utilization in breeding programmes.

The North-Eastern (NE) region of India, due to its unique eco-geographical features and high ethnic diversity, possesses at least 10,000 indigenous rice landraces which cultivated under upland, lowland and deep water conditions [24]. The impact of Green Revolution on the varietal landscape of rice in this region was insignificant, particularly in the hilly states, and the farmers still grow their heirloom varieties which not only suit their taste but also provide crop security. The current study estimates the phenotypic and genetic diversity present in a set of hill rice landraces collected from temperate to sub-temperate mountain region of the state of Arunachal Pradesh. The state of Arunachal Pradesh is situated on the eastern most corner of India sharing international borders with Bhutan, China and Myanmar. It has a geographical area of 83,743 sq. km. The state has a population density of 17 persons per sq. km, and around 68.8 % of the population is tribal [25]. The tribal communities of Arunachal Pradesh are of Tibeto-Burman linguistic origin, and there are as many as 21 tribes and 50 sub-tribes. The topography of the state is mostly hilly terrain and the agro-climate varies from tropical to alpine. The farmers follow traditional shifting or swidden cultivation (locally known as *jhum*) and sedentary agriculture. Shifting or ‘slash-and-burn’ cultivation is the earliest form of agriculture and is still practiced in vast areas of the state. About 76 % of the total cropped area in the state is under *jhum* cultivation.

Rice is the principal crop of Arunachal Pradesh and it is grown in 44.7 % of the total cropped land and accounts for 70.3 % of the total food grain production [25]. Rice productivity in the state is very low (2065 kg/ha). Here rice is grown up to an altitude of 2000 m as a rainfed crop in hill slopes of newly cleared forests (*jhum* fields), plateaus, terraces and river beds. A wide range of rice landraces were found throughout the state. Sometimes it depends on the tribal communities who inhabit the area. It was estimated that more than 70 % of the total rice varieties grown in the state are traditional cultivars or landraces having low to very low productivity [26]. Despite having low yield potential, the hill rice landraces grown in the mountains under *jhum* cultivation system possess many adaptive traits such as cold tolerance important for crop improvement programmes. Cold-tolerance in hill rice landraces was also observed in high altitude (1300–2200 m) rice areas in Nepal [27]. Similarly, in the high altitude rice areas of Arunachal Pradesh cold injury due to cool air temperature, particularly during flowering stage, is a major constraint to improving rice productivity. In general, hill rice landraces are grown on the cleared mountain sides for one year and then left fallow for 4–5 years before the farmers return to the same location (Additional file 1). The farmers usually grow few maize and millets along with rice in the same field. The hill rice landraces have been evolved by the interplay between adaptation to the harsh environment and selection imposed by the farmers who determine which varieties will be grown under a particular agro-ecological condition. Therefore, it will be interesting to study the impact of farmers’ practices and local culture on the conservation, exchange and genetic structure of traditional landrace varieties, by studying rice landraces collected from a unique ecological niche like mountain sides. There is no previous study done on genetic diversity of the hill (*jhum*) rice cultivars of Arunachal Pradesh.

In 2012, an exploration trip to three districts *viz*, East Kameng, Papum Pare and Kurung Kumey of the state of Arunachal Pradesh was conducted for the collection of hill rice landraces. These rices are direct seeded, grown on upland fields, usually on sloping hill sides (*jhum* lands), and are grown as rainfed during June to September. During the collection trip, we interviewed local farmers to gather information on significance of naming a landrace, its cultural value and popularity, and production practices of local farmers. The indigenous farmers of the surveyed region rely on gathering forest products, hunting and cultivation of rice and maize for their subsistence. In the present study, we phenotyped these rice landraces for 16 agro-morphological and grain characteristics, and genotyped them with 35 SSR markers. The specific objectives of this study were to: (i) collect information on the meanings of the variety names, special uses or properties, and important agronomic characteristics; (ii) study the agro-morphological variability; and (iii) understand the genetic relatedness and structure of these landraces.

## Methods

### Collection of hill rice

**Fig. 1:**
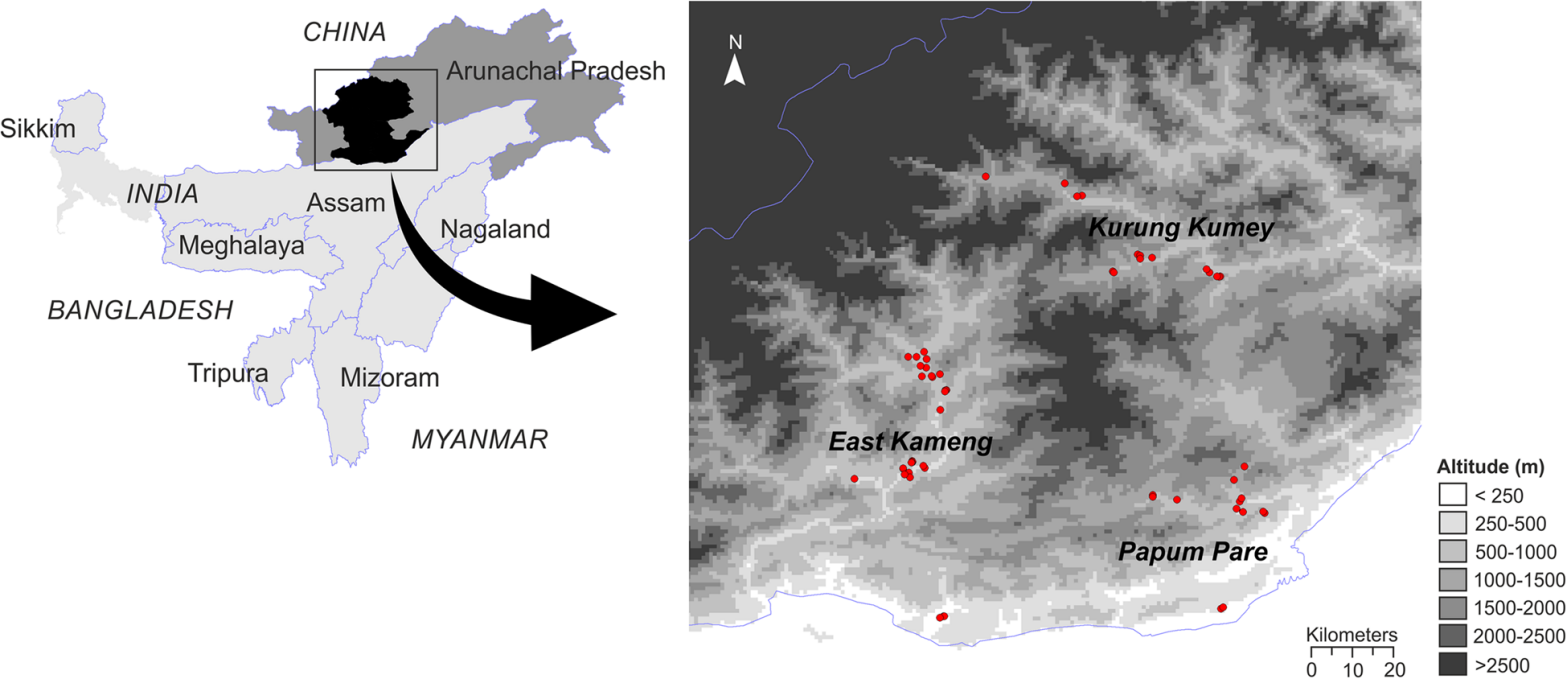
Geographic locations of the hill rice collection sites in three districts of the state of Arunachal Pradesh

A total of 67 hill rice accessions were collected from 32 villages in three districts (East Kameng, Papum Pare and Kurung Kumey) of Arunachal Pradesh, India during a collection trip from November 2–15, 2012 (Additional file 2). The geographical distribution of collected landraces is depicted in Fig. 1. The collection team, consisting of two researchers (SR and BCM) and local guide from Krishi Vigyan Kendras (Farm Science Centres) surveyed the districts and collected the landraces. In each village, seed samples were taken from the freshly harvested seed lots conserved in the farm stores. However, in some villages of Papum Pare, the seeds were collected from the standing crop. Due to poor road connectivity, the team was unable to explore very remote villages. Although the elevation of the collection sites varied from 135 m (Seijosa, East Kameng) to 1425 m (Sarli in Kurung Kumey), but the actual rice fields were much higher. Rice seeds were mostly collected from the households situated in the foothills or in the valleys. During collection, the farmers were interviewed to acquire information about the specific varieties: the meaning of cultivar name, source or origin, grain quality and special uses, disease and pest resistance, and yield.

### Plant materials

Out of these 67 hill rice accessions, altogether 64 were selected for agro-morphological and genetic characterization in this study. Information about the accessions such as name, accession number, place of collection/seed sources, farmers’ grouping of landraces and subpopulation ancestry based on STRUCTURE is given in Additional file 3. The seeds of the hill rice accessions used in this study are conserved in the National Genebank of ICAR-National Bureau of Plant Genetic Resources (NBPGR), New Delhi, India, and are publicly available for research purposes upon request with a material transfer agreement. The rice accessions were grown in 2013 and 2014 for purification. In each year, seeds from a single panicle were kept for sowing in the next year.

Three check varieties such as ‘Bhalum-1’, ‘Bhalum-3’ and ‘Sukhadhan-1’ were used during phenotypic characterization trial. ‘Bhalum-1’ and ‘Bhalum-3’ varieties are suited for cultivation under rainfed upland conditions up to an altitude of 1000 m. ‘Sukhadhan-1’ is a drought tolerant upland rice variety of Nepal. Seed of these accessions were obtained from ICAR Research Complex for North-Eastern Hill Region, Umiam, India.

A total of 15 control varieties were selected for SSR genotyping along with the hill rices (Additional file 3). These checks included five *indica* (‘IR8’, ‘IR36’, ‘Jaya’, ‘Taichung Native 1’ and ‘TKM9’), two *aus* (‘FR 13A’ and ‘Kalamkati’), three Basmati (‘Basmati370’, ‘Pakistani Basmati’ and ‘Pusa Basmati 1’), three short-grain traditional aromatic (‘Badshabhog’, ‘Kataribhog’ and ‘Mohanbhog’) and a single accession each of *tropical japonica* (‘Azucena’) and *japonica* admixture (‘Dular’). Pure seeds of these accessions were obtained from ICAR-National Rice Research Institute, Cuttack, India. These varieties were used to understand genetic grouping of the hill rice accessions, and also to act as a molecular weight reference in DNA gel electrophoresis. We did not include these varieties in phenotypic evaluation as the plant growth was very poor for most of the accessions.

To provide a reference for the rice subpopulation classification in the current study, we also used the SSR fingerprinting data of 150 rice accessions (here we denoted these as reference accessions) previously reported in the genetic diversity study by Garris et al. [9]. The band size data were obtained from ‘Gramene genetic diversity database’ (http://archive.gramene.org/db/diversity/diversity_view). We sampled 150 accessions from the originally reported 234 accessions used in Garris et al. [9] based on the recent report on open access resources for genome-wide association mapping in rice [28].

### Agro-morphological characterization

Sixty four hill rice accessions and three check varieties (‘Bhalum-1’, ‘Bhalum-3’ and ‘Sukhadhan-1’ were grown at experimental field of NBPGR, Regional Station, Umiam, Meghalaya (25.6 °N lat, 91.9 °E long and 1000 m alt) in 2015 *Kharif* season (June-November) under rainfed upland conditions. The soil of the field is an acid alfisol with a pH of 5.3, organic C 1.13 % and available N 211 kg/ha. Farmyard manure (10 tonnes/ha) was incorporated into the soil during land preparation. No chemical fertilizers and pesticides were used during crop growth period. A manual weeding was conducted to keep the crop weed free. The accessions were line-sown in 1 m x 1.5 m plots (4 lines/accession) using an augmented block design during first week of June. A spacing of ~0.25 m between the plants and 0.45 m between the rows was maintained. Phenotypic data, listed in Table 1, were recorded following the rice descriptors used by International Rice Research Institute [29]. Days required from sowing to 50 % flowering (DtF) was recorded on plot basis. The traits such as plant height (Ht), leaf length (LL), leaf width (LW), ligule length (LgL), panicles per plant (PnP) and ligule length (LgL) were recorded as the average of five randomly selected plants. Panicle length (PnL) and spikelets per panicle (SnP) were measured based on five individual measurements on the main stem. Grain and kernel morphological traits were determined from randomly sampled ten grains/kernels. The 1000-grain weight (GW) was calculated by taking weight of 100 grains from the bulk and multiplying it with 10.

**Table 1:** Range of variation in agro-morphological characteristics of hill rice landraces and the loading of the measured traits on five principal components

Traits	Group	Mean ± SD	Min	Max	%CV	*p* value	PCs				
							1	2	3	4	5
DtF	*Umte*	115.78 ± 13.05	90.00	141.00	11.27	0.110	−0.56	0.31	0.48	−0.38	−0.04
	*Tening*	120.84 ± 11.79	89.00	139.00	9.75						
Ht	*Umte*	81.11 ± 23.52	32.00	122.20	29.00	0.034	0.58	−0.39	−0.48	0.20	0.04
	*Tening*	69.86 ± 17.90	40.20	107.00	25.62						
LL	*Umte*	53.76 ± 11.04	36.00	75.00	20.53	0.053	−0.60	0.34	0.21	−0.40	−0.16
	*Tening*	59.23 ± 10.87	38.60	78.70	18.36						
LW	*Umte*	1.86 ± 0.21	1.40	2.30	11.22	0.270	−0.49	0.30	−0.24	−0.08	−0.39
	*Tening*	1.92 ± 0.25	1.40	2.40	13.10						
LgL	*Umte*	1.65 ± 0.53	0.95	2.90	31.92	0.390	0.59	−0.06	0.08	−0.43	0.16
	*Tening*	1.56 ± 0.38	0.70	2.35	24.56						
PnP	*Umte*	5.19 ± 0.97	3.50	7.20	18.76	0.410	0.11	−0.01	0.66	0.38	−0.29
	*Tening*	5.39 ± 0.97	3.50	7.30	18.02						
PnL	*Umte*	23.17 ± 2.87	16.50	27.75	12.38	0.149	0.44	−0.46	0.30	−0.09	0.48
	*Tening*	22.16 ± 2.65	17.50	28.65	11.96						
SnP	*Umte*	143.07 ± 52.53	82.50	272.50	36.72	0.234	−0.14	−0.53	0.63	0.10	0.20
	*Tening*	159.89 ± 57.13	68.00	260.50	35.73						
GrL	*Umte*	6.94 ± 0.70	5.27	8.31	10.05	0.000	0.58	0.68	0.14	0.01	0.11
	*Tening*	6.19 ± 0.65	5.17	8.32	10.44						
GrW	*Umte*	3.39 ± 0.37	2.67	3.87	10.81	0.407	−0.81	0.23	0.04	0.28	0.22
	*Tening*	3.46 ± 0.37	2.73	4.01	10.58						
GrLW	*Umte*	2.07 ± 0.31	1.73	2.97	14.81	0.003	0.87	0.32	0.08	−0.17	−0.11
	*Tening*	1.81 ± 0.34	1.40	2.91	18.87						
StLL	*Umte*	2.31 ± 0.50	1.46	3.29	21.48	0.240	0.07	0.42	−0.15	−0.43	0.60
	*Tening*	2.19 ± 0.30	1.48	2.98	13.82						
GW	*Umte*	23.03 ± 4.32	15.80	34.60	18.77	0.843	−0.25	0.49	−0.06	0.58	0.38
	*Tening*	22.85 ± 3.09	16.90	32.80	13.54						
KL	*Umte*	5.44 ± 0.46	4.48	6.24	8.36	0.000	0.70	0.49	0.16	0.34	0.04
	*Tening*	4.58 ± 0.42	3.90	6.06	9.14						
KW	*Umte*	2.9 ± 0.35	2.12	3.45	12.12	0.133	−0.84	0.11	−0.04	0.17	0.26
	*Tening*	3.02 ± 0.24	2.44	3.35	8.10						
KLW	*Umte*	1.91 ± 0.34	1.59	2.94	17.82	0.000	0.89	0.28	0.14	0.12	−0.13
	*Tening*	1.53 ± 0.25	1.23	2.48	16.11						
*Percentage Variance*							35.18	14.43	0.89	9.23	7.64
*Cumulative variance*							35.18	49.61	59.50	68.73	76.3

*DtF* days to 50 % flowering, *Ht* plant height (cm), *LL* leaf length (cm), *LW* leaf width (cm), *LgL* ligule length (cm), *PnP* panicles per plant, *PnL* panicle length (cm), *SnP* spikelets per panicle, *GrL* grain length (mm), *GrW* grain width (mm), *GrLW* grain length-to-width ratio, *StLL* sterile lemma length (mm), *GW* 1000-grain weigth (g), *KL* kernel length (mm), *KW* kernel width (mm), *KLW* kernel length-to-width ratio, *SD* standard deviation, *Min* minimum value, *Max* maximum value, *CV* coefficient of variation, *PC* principal component

### Genotyping with SSR markers

Thirty five SSR markers were designed from the ‘Gramene’ marker database (http://www.gramene.org/markers/microsat/). Information about these markers is given in Additional file 4. Extraction of total genomic DNA, polymerase chain reaction (PCR) with SSR markers and molecular size determination of amplified band were performed as described in Roy et al. [30]. During gel electrophoresis at least one control accession of ‘IR8’, ‘IR36’, ‘Taichung Native 1’, ‘FR 13A’, ‘Kalamkati’ or ‘Dular’ was used as a molecular weight reference in each gel because a reference allele size in these control accessions is available [9]. The original SSR genotype data of all rice accessions used in this study are provided in Additional file 5.

### Agro-morphological data analysis

Summary statistics such as mean, standard deviation (SD), minimum and maximum values, and coefficient of variation (CV) were determined. The mean data were standardized by deriving *Z* scores for further analyses. ANOVA was carried out to test the significance of variation between hill rice cultivar groups for different traits. Principal component analysis (PCA) was performed on the correlation matrix of the data to understand the most important variables contributing to the total phenotypic variation among the accessions. PCA represents the simplest and most commonly used multivariate method to visualize the grouping of accessions based on component loadings. Ward’s hierarchical clustering was used to understand the relationships among rice accessions based on phenotypic data. All these analyses were performed using IBM-SPSS Statistics version 20.0 [31].

### Population structure analysis

Population structure of 64 hill and 15 control rice accessions was examined using the Bayesian model-based approach implemented in STRUCTURE V2.3.4 [32]. The number of clusters (*K*) evaluated here ranged from 1 to 8. The analysis was performed using five replicate runs per *K* value, a burn-in period length of 5000, a run length of 50,000, and a model allowing for admixture and correlated allele frequency. ‘Structure harvester’ programme (http://taylor0.biology.ucla.edu) was used to determine the final *K* value(s) based on both the LnP(D) and Evanno’s Δ*K* [33]. Subsequently, ten simulations at *K* = 2–4 were then performed with a burn-in period of 10,000 and a run length of 100,000. The membership coefficient from the run with the lowest likelihood value was used to assign each accession to the *K* = 1 to 4 subpopulations based on the estimated membership coefficients.

### Summary statistics and genetic diversity analysis

The average number of alleles per locus (AN), major allele frequency (MAF), gene diversity (*H*_*e*_), heterozygosity (*H*_*o*_) and polymorphism information content (PIC) were calculated using PowerMarker V3.25 [34]. Average allelic richness (*R*_*s*_) and Wright’s fixation index (*F*_*st*_) values were calculated using FSTAT V2.9.3.2 [35]. The molecular variance of subpopulations and accessions within the subpopulations were calculated using an Analysis of Molecular Variance (AMOVA) approach in GenAlEx V6.5 [36]. Separate analyses were conducted by classifying the rice accessions into: districts, farmers’ classified groups and STRUCTURE subpopulations. The statistical test of the differences in allelic count among different groups with variable numbers of accessions was conducted in R [37] using FPTestR, the R version of FPTest method reported in Fu et al. [38]. The FPTestR package (communicated for publication) was kindly provided by Dr Yong-Bi Fu, Plant Genetic Resources of Canada, Agriculture and Agri-Food Canada. The allele frequency data from PowerMarker was used to export the data in binary format (1/0) for analysis with NTSYS-pc V2.2 [39]. A neighbour-joining (NJ) cluster diagram was constructed with the NJOIN sub-programme using the genetic dissimilarity matrix calculated in SIMGEND sub-programme with Nei72 coefficient. To summarize the patterns of variation in multi-locus dataset, principal coordinate analysis (PCoA) was performed in GenAlex software using the genetic distance matrix among the accessions.

### Combined analysis of hill and reference accessions

Cluster analysis was conducted on hill and reference accessions to understand the grouping of hill rices in respect to different genetically defined groups (*indica*, *aus*, *aromatic*, *tropical japonica* and *temperate japonica*). The reference set also included some ‘admixture’ accessions. For clustering, a pair-wise genetic distance matrix was calculated in PowerMarker following C. S. Chord distance method [40]. The NJ method was used for phylogenetic reconstruction. The unrooted NJ tree was visualized using Dendroscope V3 [41]. Pair-wise *F*_*st*_ and allelic differences (FPTest) between the groups were calculated as stated earlier.

## Results

### Variability in agro-morphological traits

Based on farmers’ classification of the hill rices, the collected rice germplasm was categorized into two cultivar types: (i) *umte* (*um* = rice; *te* = large)*,* large grained-late maturing rice, and (ii) *tening*, small grained-early maturing types (Additional file 3). The *umte* cultivars are high yielding and good to taste. In this study, we have assessed the agro-morphological and genetic diversity between these two groups.

Descriptive statistics for 16 agro-morphological characteristics in *umte* and *tening* groups of hill rice are given in Table 1. The variation observed between two groups was significant for the traits evaluated such as plant height (Ht), leaf length (LL), grain length (GrL), grain length-to-width ratio (GrLW), kernel length (KL) and kernel length-to-width ratio (KLW). There was no significant difference in days to 50 % flowering (DtF) between gropus. The range of variation in DtF in both the groups was similar, and interestingly *tening* group showed higher average value of DtF (120.8 d) than *umte* (115.8 d). However, the difference was prominent for grain and kernel characteristics. The *umte* accessions had longer grains with an average value of 6.9 mm than that of *tening* (6.2 mm). Similarly, the value of KL in *umte* (5.4 mm) was higher than that recorded in *tening* (4.6 mm). Both the highest values for GrLW and KLW were recorded in *umte*. On average, the plants of *umte* accessions (81.1 cm) were taller than the *tenings* (69.9 cm). The *tenning* accessions produced larger leaves (59.7 cm).

**Fig. 2:**
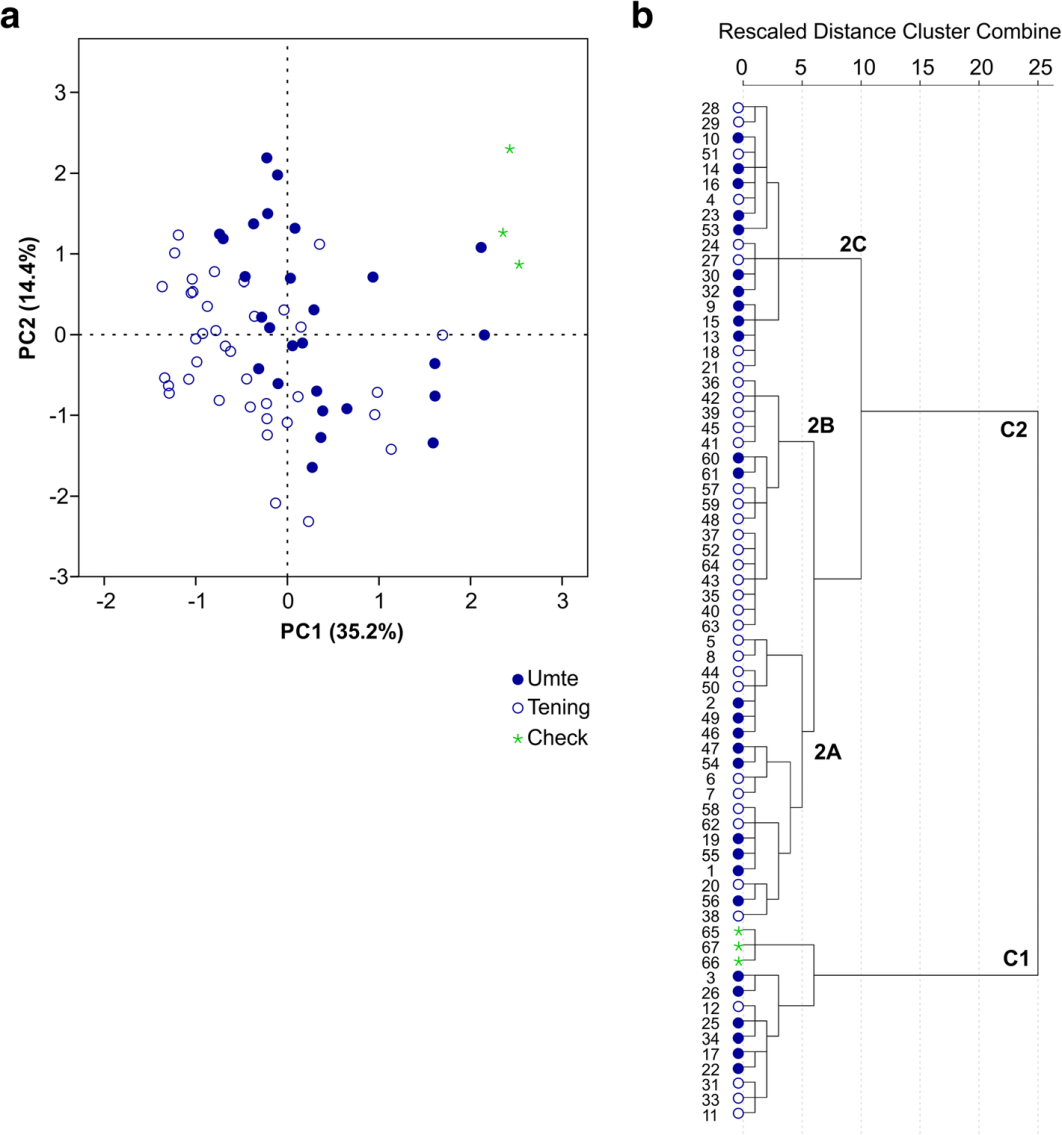
Grouping of the hill rice landraces based on 16 agro-morphological characteristics. **a** Principal component bioplot constructed using first two principal components; **b** Ward’s hierarchical cluster clustering. The serial numbers of rice accessions are given in Additional file 3. *Umte* and *tening* accessions are represented using filled and hollow circles, respectively

Principal component analysis (PCA) using 16 traits produced five principal components (Eigenvalue > 1) which cumulatively accounted for 76.4 % of the total phenotypic variance (Table 1). The first PC explained 35.2 % of the total variance and the traits with high positive loadings were KLW, GrLW, KL, GrL, LgL (ligule length) and Ht. The PC2 explained an additional 14.4 % of the total variance. The traits such as GrL, KL and 1000-grain weight (GW) showed high positive loadings. The variation in panicles per plant (PnP), spikelets per panicle (SnP) and DtF was largely explained by PC3 (Table 1). The PCA biplot with PC1 and PC2 scores showed that the accessions belonging to *umte* and *tening* groups clustered separately based on the phenotypic traits (Fig. 2a). The *umte* accessions having higher values for GrL, GrLW, KL, KLW, Ht and PnL, positioned at the right half of the biplot. The *tening* accessions occupied the left half of biplot mainly because of higher values for the traits such as SnP, GrW and KW. The three check varieties used in phenotypic characterization were grouped separately from most of the hill rice accessions.

Ward’s hierarchical cluster analysis revealed a similar grouping of accessions as found in PCA (Fig. 2b). Overall, 67 accessions were divided into two groups. All check varieties formed a distinct cluster (C1) along with ten hill accessions. The C2 was further divided into two sub-clusters. There was no clear grouping of *umte* and *tening* accessions, except a sub-group (C2B) of 15 *tenning* accessions which had highest values for KW, GrLW, DtF, LL and leaf width (LW).

### SSR variation

The summary statistics of 35 SSR markers are given in Additional file 4. Overall, 297 alleles were detected at the 35 SSR markers, ranging from 2 alleles (RM338) up to 21 alleles (RM259), with an average of 8.49 alleles across the loci. The gene diversity or expected heterozygosity (*H*_*e*_) ranged from 0.41 (RM55) to 0.94 (RM259) and the average observed heterozygosity (*H*_*o*_) was 0.051. Allelic richness (*R*_*s*_) varied from 2.0 (RM338) to 14.6 (RM259) with an average value of 6.04 across the study. The polymorphism information content (PIC) values ranged from 0.37 (RM338 and RM507) up to 0.93 (RM259) with an average of 0.65.

### Genetic structure and subdivision of hill rice accessions

**Fig. 3:**
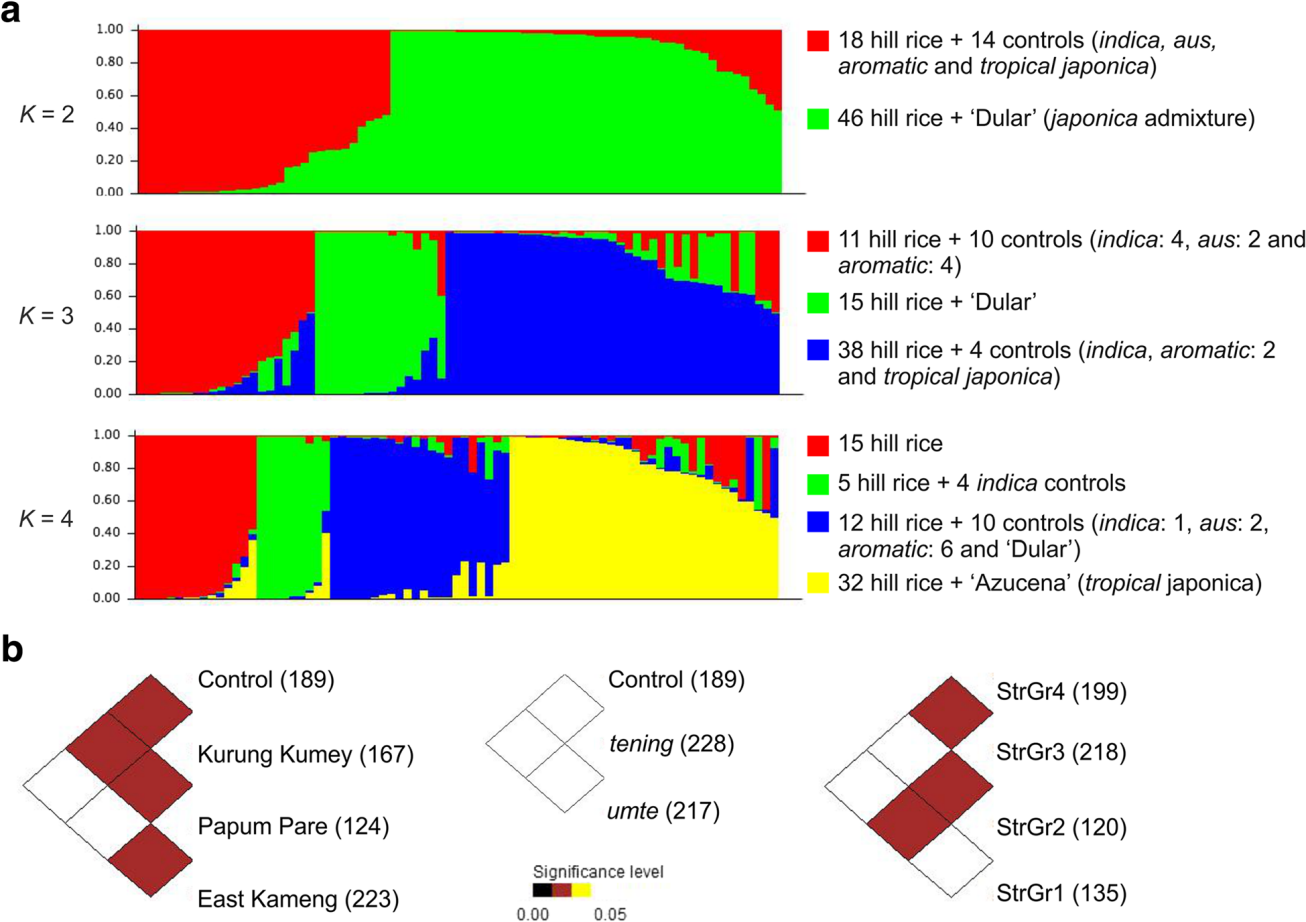
**a** Model-based population structure of 79 rice accessions at *K =* 2 to 4; **b** Pair-wise tests of allelic differences between different groups of the hill rice accessions. The number of observed alleles in each group is given in the parenthesis

The inferred population structures are given in Fig. 3a and Additional file 3. By comparing LnP(D) and Evanno’s ∆*K* values by increasing *K* from 1 to 8, we found that LnP(D) values increased with *K*, with the highest log likelihood score at *K* = 2, while ∆*K* value was also highest at *K* = 2 (Additional file 6). No peak of ∆*K* was evident at *K* > 3. This indicated that these 64 hill and 15 control accessions had a genetic structure of two subpopulations, supporting occurrence of two subspecies: *Indica* and *Japonica*. However, we have presented the structures at *K* = 2 to 4, to check for biological relevance. At *K* = 2, group 1 had 32 accessions including 11 control varieties belonging to *indica*, *aus* and *aromatic*. Altogether, 10 admixture accessions (having <80 % of inferred ancestry from any one group) including ‘Azucena’, ‘TKM9’ and ‘Pakistani Basmati’ were identified in this group. Group 2 consisted of a total of 47 accessions including ‘Dular’, a *japonica* admixture variety, as admixture. At *K* = 3, ten control varieties (*indica*, *aus* and *aromatic*) formed group 1 along with 11 hill accessions. Group 2 has 15 hill rices along with ‘Dular’ as admixture. A majority of hill rices (37 accessions) were included in group 3 along with the control varieties such as ‘Azucena’, ‘TKM9’, ‘Pakistani Basmati’ and ‘Mohanbhog’. The subpopulation structure in these rice accessions was more clear at *K* = 4. The four subpopulations (StrGr1 to 4) had a population-specific *F*_*st*_ value of 0.33, 0.33, 0.06 and 0.20, respectively, with an average of 0.23, indicating a moderate population structure. At this level, *indica* control varieties were separated from *aus* and *aromatic* varieties. The aromatic *tropical japonica* variety ‘Azucena’ showed separate grouping along with 50 % of hill accessions. A group of 15 hill accessions formed a distinct group (StrGr1). At *K* = 4, a total of 25 accessions were identified as an admixture.

**Fig. 4:**
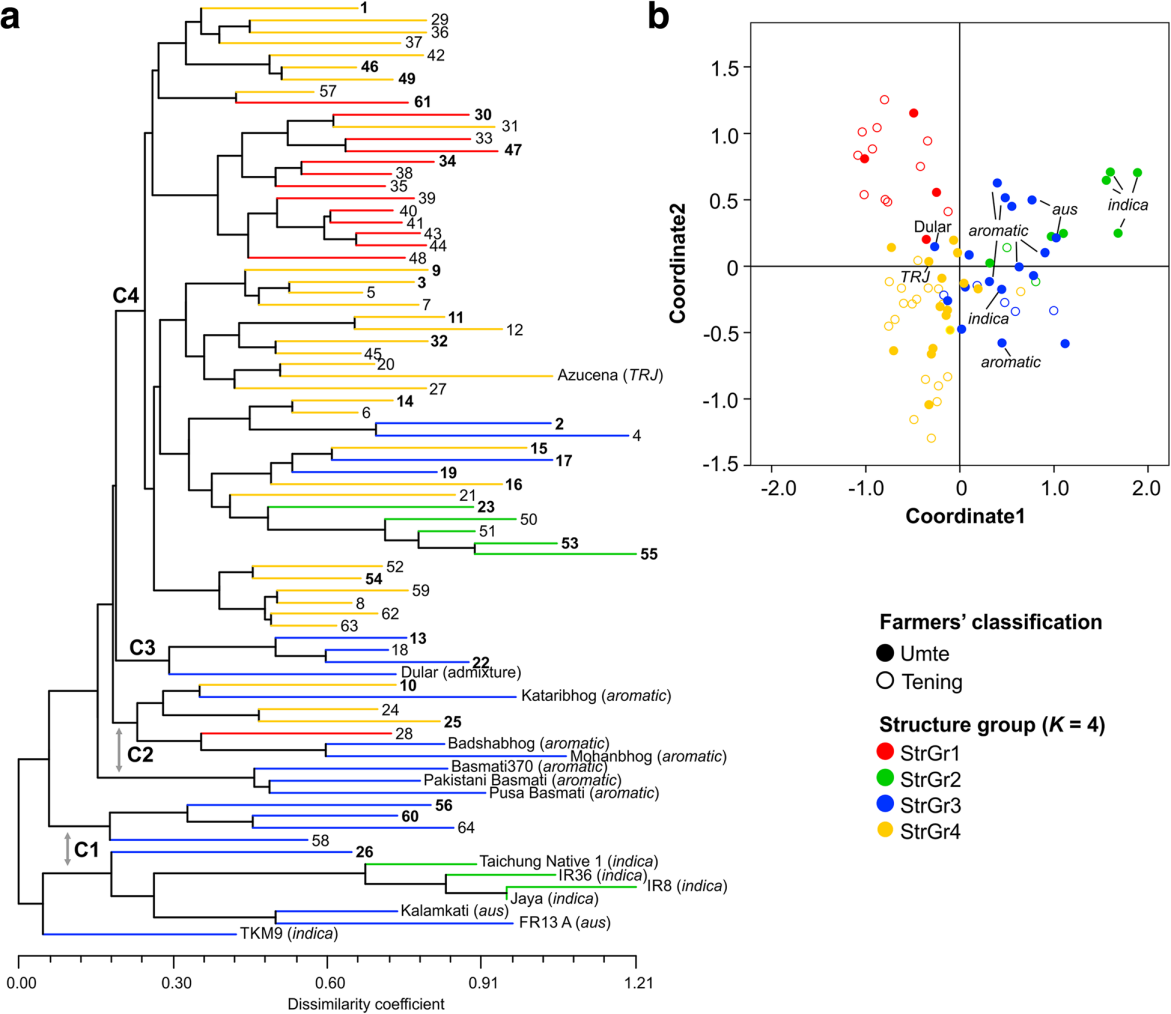
Genetic grouping of 64 hill rice landraces in relation to the control varieties. **a** Neighbour-joining tree based on C. S. Chord genetic distance. The rice accessions are numbered as per Additional file 3. Bold-faced font represents *umte* accessions; **b** Principal coordinate analysis: *Umte* and *tening* accessions are represented using filled and hollow circles, respectively. In both (**a** and **b**), the accessions have been colour coded as per STRUCTURE results at *K* = 4

The neighbour-joining (NJ) analysis of pair-wise genetic distances detected four major clusters (C1-C4; Fig. 4a). These clusters showed varied regional heterogeneity. However, the hill accessions collected from Papum Pare district grouped in cluster 4. Cluster 1 was consisted of all *indica* and *aus* controls, along with four hill accessions collected from Kurung Kumey and a single accession from East Kameng. Cluster 2 grouped all *aromatic* control varieties and four hill accessions collected from East Kameng. The rest hill rices grouped either with *tropical japonica* variety ‘Azucena’ (53 accessions) or with ‘Dular’ (3 accessions). The cluster membership of each accession is given in Additional file 3. Upon labelling the accessions according to their inferred STRUCTURE ancestry, we did not find exact match between the results of NJ cluster and STRUCTURE (*K* = 4). However, the PCoA results were consistent with STRUCTURE (Fig. 4b). In PCoA, first two coordinates clearly separated four STRUCTURE subpopulations. The *indica*, *aus* and *aromatic* control varieties formed a distinct group in the PCoA plot. Note that the farmers’ classification of hill rices into *umte* and *tening* group based on morphological traits was not supported by any of the analyses. Taken together, all three approaches demonstrated that the majority of these hill rice accessions are *Japonica* or admixture among the groups within either *Japonica* or *Indica*.

### Genetic diversity

The rice accessions displayed different pattern of SSR variation with respect to district, cultivar group and STRUCTURE grouping (Table 2). The AMOVA revealed significant variations (*p* = 0.001) among and within subpopulation groups corresponding to districts, STRUCTURE groups and overall hill and control accessions (Additional file 7). However, a non-significant (*p* = 0.089) 0.8 % of the total variation among *umte* and *tening* groups was recorded. Considering the districts, the highest genetic diversity was observed in East Kameng accessions (0.65) with a mean of 6.31 alleles per locus. The lowest genetic diversity was recorded in the accessions from Papum Pare (0.50) with a mean of 3.54 alleles per locus (Table 2). Between *umte* and *tening* groups, the genetic diversity and mean number of alleles per locus (AN) was identical. The *umte* group had the highest PIC value in spite of having lower number of accessions. Among four STRUCTURE subpopulations, the highest genetic diversity was observed in StrGr3 (0.69) with an AN of 6.26, followed by StrGr4 (0.59) with an AN of 5.69. Similar trends were observed for PIC and allelic richness (*R*_*s*_) values. The overall genetic diversity in hill and control accessions was at par, but the hill rices had the highest *R*_*s*_ (5.47) and AN (7.4). Note that, despite having only 15 accessions, the control group recorded the highest genetic diversity and PIC. This may be due to inclusion of diverse rice control varieties from different genetic groups.

**Table 2:** Genetic diversity statistics of hill rice landraces at different sub-population levels

Sub-population	n	AN	MAF	*R*_*s*_	*H*_*e*_	PIC
*Districts*						
East Kameng	34	6.31	0.49	5.32	0.65	0.61
Papum Pare	13	3.54	0.62	3.51	0.50	0.45
Kurung Kumey	17	4.77	0.51	4.54	0.61	0.56
*Farmers’ classified groups*						
*Umte*	27	6.14	0.46	5.54	0.67	0.63
*Tening*	37	6.51	0.50	5.30	0.63	0.59
*STRUCTURE* *sub-populations (K = 4)*						
StrGr1	15	3.77	0.61	3.51	0.51	0.46
StrGr2	9	3.49	0.56	3.49	0.53	0.48
StrGr3	22	6.26	0.44	5.20	0.69	0.65
StrGr4	33	5.69	0.54	4.42	0.59	0.55
*All hill accessions*	64	7.40	0.47	5.47	0.66	0.62
*Controls*	15	5.40	0.43	5.28	0.68	0.64

*n* number of accessions, *AN* average number of alleles per locus, *MAF* major allele frequency, *R*_*S*_ average allelic richness. *H*_*e*_, expected heterozygosity, *PIC* Polymorphic information content

The number of alleles in a sample (allelic count or allelic richness) is an important measure of diversity. Pair-wise tests of allelic differences between different groups of accessions are given in Fig. 3b. Allelic counts of Papum Pare and Kurung Kumey was significantly different with that of the control. The allelic differences between the districts were also significant, except the case between East Kameng and Kurung Kumey. No significant allelic differences were noted between *umte* and *tening*, or between controls and either of the two groups. Among the four STRUCTURE subpopulations, allelic count of StrGr3 was significantly different with that of StrGr1 and StrGr2 (Fig. 3b). Pair-wise allelic difference between StrGr3 and StrGr4 was also significant.

Pair-wise estimates of *F*_*st*_ values among and between subpopulations are given in Additional file 8. The level of differentiation between *umte* and *tening* was very low (0.008). Overall, the rice accessions from three districts had a low level of differentiation with *F*_*st*_ values ranging from 0.043 to 0.126. Among the STRUCTURE groups, the greatest differentiation was observed between StrGr1 and 2 (0.290), and the lowest was between StrGr3 and 4 (0.086).

### Genetic grouping in relation to reference rice accessions

**Fig. 5:**
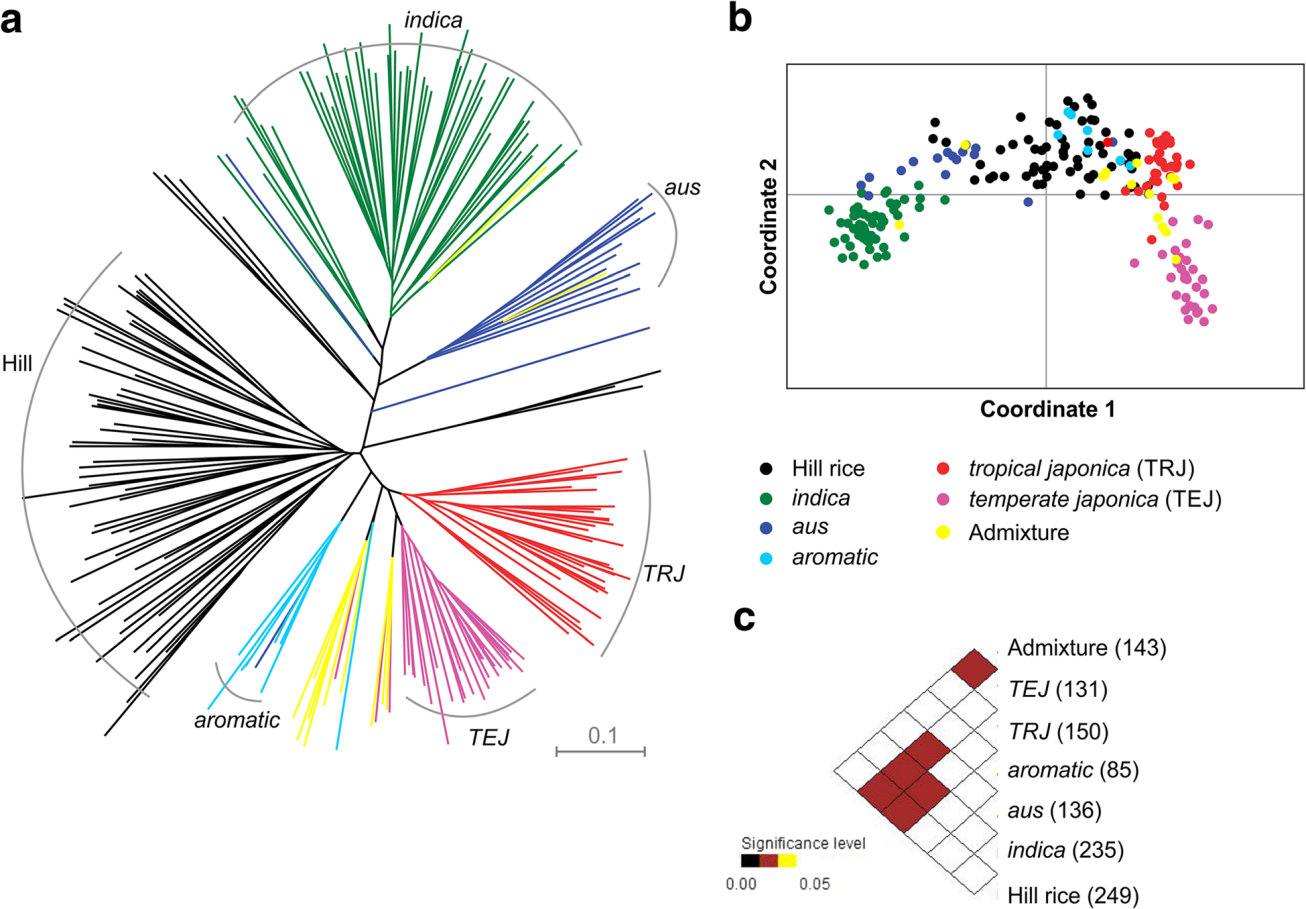
Genetic grouping of 64 hill rice accessions in relation to the reference rice cultivars. **a** Neighbour-joining tree based on C. S. Chord distance; **b** Principal coordinate analysis; **c** Pair-wise tests of allelic differences between different groups of rice accessions. The number of observed alleles in each group is given in the parenthesis

We sampled 150 diverse rice cultivars representing all five major groups identified in previous studies [9, 28] to analyze the genetic grouping of hill rice landraces included in this study. The reference dataset of 33 SSR markers (Additional file 4), common with the present study, was independently re-analysed to check the grouping of the accessions into five clusters as originally obtained by using a total of 169 SSRs [9]. Phylogenetic reconstruction confirmed a similar grouping of 150 reference rice accessions into five genetic clusters corresponding to *indica*, *aus*, *aromatic*, *tropical japonica* and *temperate japonica* (Additional file 9). In both NJ clustering and PCoA, the majority of the hill rice accessions were grouped in close association with *aromatic* and *tropical japonica* varieties (Fig. 5a and b, respectively). We also included a few *japonica* admixture varieties in the reference dataset to see their grouping in comparison to the hill rices. However, all the admixture varieties occupied a separate place in the genetic cluster. Difference in allelic richness was significant between hill and both *tropical* and *temperate japonica* (Fig. 5c). The pair-wise estimates of *F*_*st*_ values based on allelic difference revealed that hill rice accessions had the highest degree of differentiation with *temperate japonica* (0.235), followed by the *indica* (0.231) (Additional file 8). The lowest *F*_*st*_ value was recorded between hill and admixture accessions (0.095).

## Discussion

Our characterization of hill rice germplasm of North-Eastern region of India has revealed that a diverse array of rice landraces was grown by the farmers which traditionally classified as *umte* and *tenning* based on grain morphology and crop duration. These two groups of hill rice germplasm varied significantly for the traits such as plant height, leaf length, grain/kernel length and grain/kernel length-to-width ratio, but non-significant genetic differentiation between the two groups were observed. Population genetic analysis showed that these hill rices were mostly *japonica* or admixture among the subpopulations of *Indica* or *Japonica*. These results are useful for understanding the agro-morphological variability, genetic diversity and structure of hill rice landraces, and significant for utilization of hill rices in crop improvement programmes. Our study also provides baseline information for planning in-situ conservation of the hill rice landraces, which are adapted to local environments in harmony with cultural preferences of the farmers.

### Hill rice landrace

The traditional names of rice cultivars are generally found to be a valuable concept in describing the landraces worldwide. In the present study, the names of rice cultivars varied among the districts. Few rice accessions ‘Taba’, ‘Kilung’ and ‘Sarpung’ were cultivated in all three districts. The farmers in Arunachal Pradesh most often name the rice landraces after their place of origin. For instance, ‘Nepali dhan’ is originated in Nepal. Likewise, ‘Manipuri dhan’ and ‘Naga dhan’ are from the state of Manipur and Nagaland, respectively. Rice landraces often named after a community/tribe. For example, ‘Taba dugu’ and ‘Adi’ named after ‘Taba’ and ‘Adi’ communities of Arunachal Pradesh, respectively. During collection trip the farmers expressed that hill rice cultivars generally taste better than the low land cultivars, and thus used for making local delicacies like *pitha* and sweets. Therefore, knowledge about the local names of rice landraces is useful for understanding their origin as well as grain quality and other characteristics. The traditional rice landraces possess wide genetic variability valuable for rice breeding [42, 43]. The broad genetic base of traditional rice cultivars is suitable for subsistence farming practiced by the farming communities. Rice germplasm collected from geographically isolated and ecologically important sites need to be characterized both at phenotypic and genetic levels to understand the interaction and genetic controls for traits influencing adaptation of rice crop to harsh environments, and to identify sources for important adaptive traits [44]. In Nepal, the rice accessions sampled from mid- and high-hill sites were found to possess tolerance to cold stress. However, interestingly rice diversity in the high-hill regions was lower than those of mid-hill and low-hill sites [45]. It was proposed that rice diversity in the mid-hills is linked with the range in altitude in these regions that results in great environmental heterogeneity and diverse agro-ecosystems, and great diversity in the socio-economic structure of the farming communities [46].

### Agro-morphological differentiation

The tribal farmers of Arunachal Pradesh usually classify hill rice cultivars on the basis of grain morphology and/or quality characteristics, in addition to other agronomic traits such as crop duration. In the present study, we have assessed the variability in agronomic and grain/ kernel morphological traits to understand the morphological factors differentiating the two groups (*umte* and *tening*). Our analysis apparently showed that two groups of hill rice are mainly differentiated by the traits such as grain/kernel length and length-to-width ratio. We did not find significant variation in days to flowering between *umte* and *tening*. However, this is a key trait considered by the farmers in categorizing the hill rices. The non-significant variation in days to flowering in the present study may be due to change in crop growing conditions including soil and other environmental factors. We also performed a step-wise canonical discriminant analysis (CDA) taking *umte* and *tening* as priori groups to identify the most important discriminating traits contributing to the grouping of hill rices. Kernel length and plant height were found to be the best predictors for classifying the cultivar groups (results not shown). In both PCA and cluster analysis, we found that the accessions with similar names formed close groups, indicating the consistency in naming the cultivars by traditional farmers. However, in a different study with 130 indigenous rice accessions from a neighbouring state (Nagaland) in NE India, we found inconsistency in naming the cultivars [47]. Similar observation was also reported in upland rices of Nepal [48]. The grouping of hill rice accessions in both PCA and Ward’s cluster did not follow geographical origin for a genotype. Similar trend was also recorded in previous studies [47, 49]. The average number of panicles per plant in hill rice accessions of Arunachal Pradesh (5.30) is lower than those reported in rice landraces from Nagaland state (7.9) [47] and rice cultivars adapted to high-altitude environments in Nepal (8.0) [48]. Extremely strong selection pressure for adaptation to the harsh high-altitude environments and direct seeding method of planting could have limited the number of productive tillers in hill rice germplasm.

### Genetic diversity and structure

In the present study, we have used 35 SSR markers for genotyping rice landraces. A majority of these markers were chosen from the panel of 50 standard SSR markers recommended for rice diversity analysis by the Generation Challenge Programme by CGIAR. Among these SSRs, 33 markers also represent a subset of 169 markers originally used by Garris et al. [9]. Grouping 150 reference rice accessions from Garris et al. [9] using these 33 SSRs, was nearly the same. This suggests that the 35 SSR markers used in the present study can sufficiently resolve the genetic structure in rice.

Generally, the rice varieties grown under upland culture in the hill areas of Southeast Asia as a component of shifting cultivation are *japonicas* [1]. Earlier studies also indicated that a majority of upland rice landraces grown in upland fields are *japonica* [18]. However, upland *indica* cultivars were also recorded in different studies [20, 50]. An earlier study targeting *indica-japonica* classification of Asian rice ecotypes and Japanese rice cultivars proposed that lowland rices can be clearly classified into *indica* or *japonica*, while, upland cultivars cannot [51]. In the present study, we attempted to classify the hill rice accessions by grouping them in relation to the control varieties. The STRUCTURE analysis revealed that considering the optimal grouping of the genotypes into two clusters, 79 rice accessions can be divided in to two broad groups: *Indica* and *Japonica*. Among the hill rices, only 18 accessions grouped with *indica* and *aus* control varieties, suggesting that the majority of hill rices are *Japonica*. Based on the STRUCTURE grouping at *K* = 4, 50 % of hill rice accessions were identified as *japonica*. The number of admixed individuals at *K* = 4 was considerable higher than that at *K* = 2, which suggests that the majority of hill rice cultivars are derived from admixture among the groups within either *Indica* or *Japonica* rather than between two subspecies. Similar findings were also reported in the Chinese rice collection [14]. Genetic grouping of rice accessions in both NJ cluster and PCoA also supported the STRUCTURE results. The genetic grouping of rice accessions in this study did not support the farmer’s classification of hill rices. This discrepancy may be due to the limited number of SSR loci used in diversity analysis. These 35 SSR markers might reflect only a small part of the genotype-phenotype association of otherwise complex traits like grain and kernel length and shape, which are actually attributed to multiple loci with small effect [52, 53]. Similar observations were also reported in upland rice accessions of Nepal when analysed with 36 SSR markers [48], and in a set of 91 rice accessions from Eastern and NE India using 23 SSR markers [54].

In the present study, the genetic grouping of hill rices based on SSR data was also inconsistent with the geographical distribution (district-wise) and naming of rice cultivars. We cannot explain why geographical distribution did not match with genetic relationship among accessions. Seed exchange between the villages might be the reason. We noted that the farmers in Arunachal Pradesh most often gift seeds of good rice varieties during wedding ceremonies. It has been observed that the landraces with the same name collected from different villages/district are not always genetically identical. Similar observations were also reported in other studies [18, 48]. Therefore, during the germplasm collection, cultivars with the same names need to be collected as each accession is inherently valuable.

The current hill rice accessions revealed overall genetic diversity (*H*_*e*_ = 0.66; PIC = 0.62) similar to the 107 aromatic rice cultivars of NE India (*H*_*e*_ = 0.67; PIC = 0.62) [30]. Slightly lower genetic diversity was reported in a subset of 26 rice accessions collected from NE India (PIC = 0.57) [54]. The genetic diversity in the current hill rice accessions was considerably higher than that found in high-altitude rice cultivars of Nepal (PIC = 0.17) [48]. Although allelic diversity indices could be used as indicators of genetic variation in germplasm collection, such estimates are relative and largely depend on the number of polymorphic loci and relatedness of genotypes included [55]. The diverse nature of Arunachal Pradesh hill rices may be a reflection of the prevalent diverse agro-climatic, ethno-cultural and eco-geographical features of the state. Interestingly, the genetic diversity between the *umte* and *tening* groups of hill rice was at par, and pair-wise allelic difference was non-significant. This is perhaps due to similar genetic makeup of the accessions of two morphological groups. The genetic diversity estimates among four STRUCTURE subpopulations were higher for group 3 and 4 (*H*_*e*_ = 0.69 and 0.59, respectively), which can be categorized as *japonica* groups. However, the gene diversity in nine accessions of groups 2 was slightly lower (*H*_*e*_ = 0.53). This group was characterized by *indica* accessions, and higher genetic diversity of these accessions supports the fact that *indica* accessions are more diverse than *japonica* accessions [15, 56].

## Conclusions

The present research, although with a limited number of SSR markers, indicated that hill rices were mostly *japonica* or admixture among the subpopulations of *Indica* or *Japonica*. Both FPTest and *F*_*st*_ results indicated that the hill accessions are genetically diverse from *temperate japonica* and *indica*. The rice germplasm prevailing in NE India has great importance in studying the evolution and population genetics of cultivated rice. This region is considered to be the domestication origin of *Indica* rices [1]. While, the adjoining Southern China is the proposed origin of *Japonica* rice [1, 57]. Therefore, the bordering region of China and India can be considered as the differentiating zone of the two subspecies. Many studies in the past have reported the occurrence of diverse rice ecotypes, belonging to various genetic groups of rice, in NE India and Bangladesh [58]. Prevalence of *Japonica* rices has also been recorded in NE Indian rice germplasm [30, 59]. The hill rice germplasm reported in the present study may be a valuable resource for studying *Indica-Japonica* evolution.

The findings presented here also have some implications for the utilization and conservation of hill rice germplasm. First, considerable diversity, both in agro-morphological and genetic terms, has been maintained by the traditional farmers. This is most likely the result of maintaining different sets of landraces in different regions in response to local heterogeneities in climate and soil type, as well as indigenous culture, as demonstrated by Fu et al. [60] in Thai cassava farming. However, due to increased susceptibility to pests and diseases, some landraces have become obsolete. Second, our findings also are encouraging for on-farm conservation programme in the study location, along with the ex situ conservation efforts. The genetic variation among the cultivars from different districts was not significant, which indicates that on farm conservation programme can be initiated in any of the three districts of Arunachal Pradesh. Third, significant variations for many agro-morphological traits can be exploited in the rice breeding programmes. The information on genetic distance among the landraces could be used to guide the selection of valuable germplasm in rice breeding.

## Abbreviations

AMOVA, analysis of molecular variance; AN, average number of alleles per locus; ANOVA, analysis of variance; CDA, canonical discriminant analysis; CGIAR, Consultative Group on International Agricultural Research; CV, coefficient of variation; ICAR, Indian Council of Agricultural research; MAF, major allele frequency; NJ, neighbour joining; PCA, principal component analysis; PCoA, principal coordinate analysis; PCR, polymerase chain reaction; PIC, polymorphism information content; SD, standard deviation; SSR, Simple sequence repeats

## Additional files

Additional file 1: Hill rice cultivation in the state of Arunachal Pradesh. a *Jhum* fields after rice harvesting; b Harvesting of hill rice by a lady of *Nyishi* community in Papum Pare district; c rice granary containing harvested and stored grain; d An overview of grain morphology of the hill rice accessions collected for the study. (PDF 2308 kb) Additional file 2: Arunachal Pradesh villages where hill rice accessions were collected. (DOC 39 kb) Additional file 3: Names, accession number, classification and genetic structure of 64 hill rice landraces and control varieties used in SSR fingerprinting. (DOC 135 kb) Additional file 4: Details and summary statistics of 35 SSR markers used in the study. (DOC 73 kb) Additional file 5: The original genotype data of hill rice accessions along with the control varieties. (XLSX 38 kb) Additional file 6: Determination of the best *K* value(s) based on mean LnP(D) over five runs for each *K* value, and rate of change in the log probability of data between successive *K* values (∆*K*). (PDF 1363 kb) Additional file 7: Analysis of SSR molecular variance. (DOC 32 kb) Additional file 8: Pair-wise *F*_*st*_ values for different sub-populations. (DOC 58 kb) Additional file 9: Phylogenetic reconstruction of 150 reference rice accessions (Garris et al., 2005) based on 33 SSR markers data. (PDF 1358 kb)
