# Supplementary material for: Genetic diversity and structure in hill rice (Oryza sativa L.) landraces from the North-Eastern Himalayas of India
Source: BMC Genet. 2016 Jul 13;17:107. doi: 10.1186/s12863-016-0414-1 (PMC4944464; doi:10.1186/s12863-016-0414-1)
Supplement: Additional file 1: — Hill rice cultivation in the state of Arunachal Pradesh. a Jhum fields after rice harvesting; b Harvesting of hill rice by a lady of Nyishi community in Papum Pare district; c rice granary containing harvested and stored grain; d An overview of grain morphology of the hill rice accessions collected for the study. (PDF 2308 kb) [file 12863_2016_414_MOESM1_ESM.pdf]

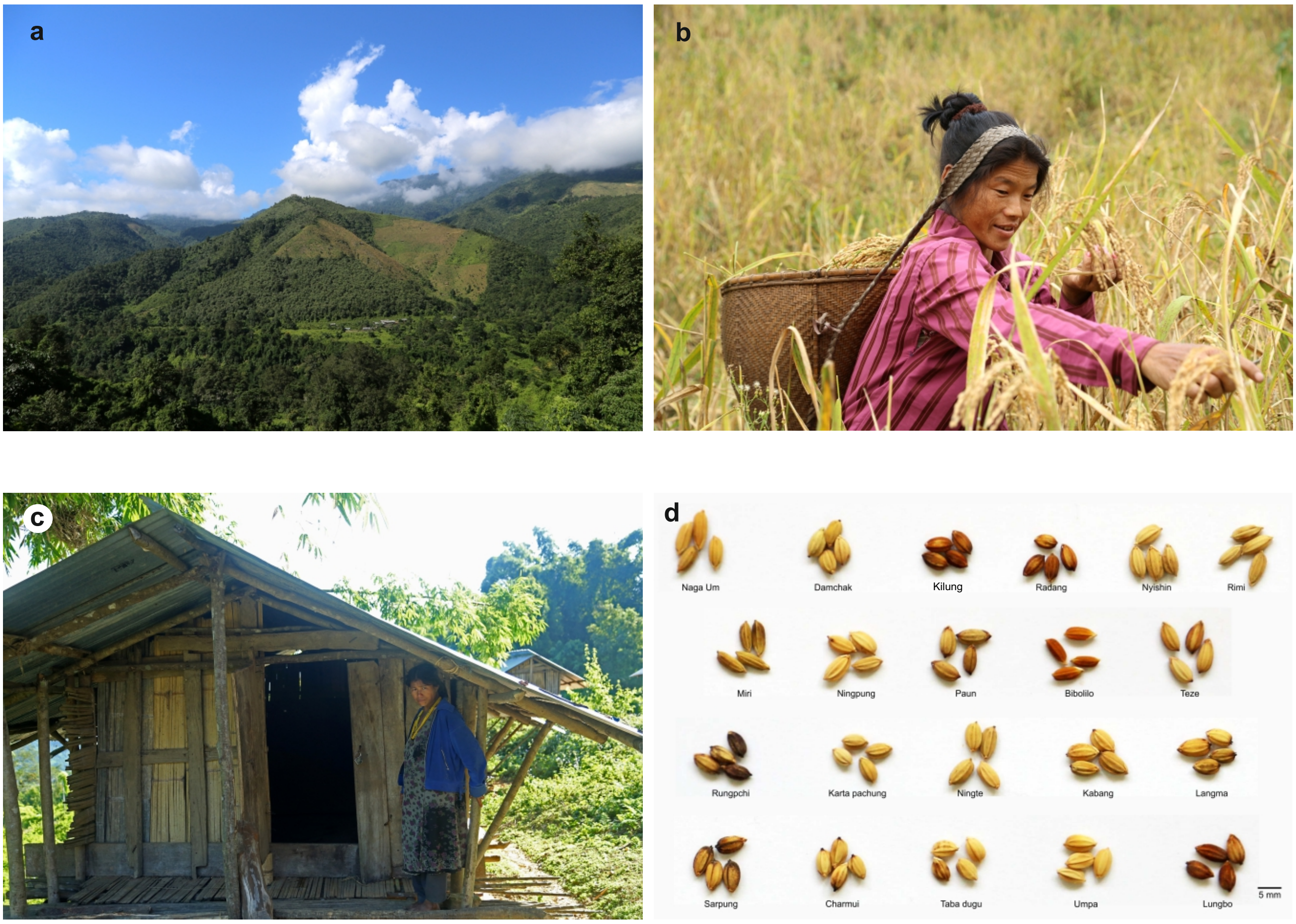

Additional file 1: Hill rice cultivation in the state of Arunachal Pradesh. **a** *Jhum* fields after rice harvesting; **b** Harvesting of hill rice by a lady of *Nyishi* community in Papum Pare district; **c** rice granary containing harvested and stored grain; **d** An overview of grain morphology of the hill rice accessions collected for the study
