# Supplementary material for: Genetic diversity and structure in hill rice (Oryza sativa L.) landraces from the North-Eastern Himalayas of India
Source: BMC Genet. 2016 Jul 13;17:107. doi: 10.1186/s12863-016-0414-1 (PMC4944464; doi:10.1186/s12863-016-0414-1)
Supplement: Additional file 2: — Arunachal Pradesh villages where hill rice accessions were collected. (DOC 39 kb) [file 12863_2016_414_MOESM2_ESM.doc]

**Additional file 2:** Arunachal Pradesh villages from where hill rice accessions were collected

| **District** | **Circle/Mandal** | **Village** | **Elevation range (m)^a^** | **Accessions collected^b^** | **Topography** |
| --- | --- | --- | --- | --- | --- |
| East Kameng | Bana | Bana, Kafla, | 414-456 | 3 | Foothill |
|  | Seppa | Fengche, Jayanti, Neping, Kampu, Tezu, Londa, Riga Kemp, Pachi Kemp, | 404-936 | 22 | Hill |
|  | Pakke | Pakke, Yardang, | 569, 861 | 2 | Hill |
|  | Seijosa | Mobuso, Mobuso (I), Bali | 135-188 | 7 | Foothill and valley |
| Papum Pare | n/a | Satang, Sagali, Kheel, Toru (Rumi) | 407-930 | 8 | Hill |
|  | Ampuli | Sankang | 805-857 | 3 | Hill |
|  | n/a | Depo, Pith (Lower Subansiri) | 1140, 1411 | 2 | Hill |
| Kurung Kumey | Old Palin | Lumba, Langba, Chouba | 970-1287 | 6 | Hill |
|  | Sangram | Pagba (I) | 1132 | 1 | Hill |
|  | Niapin | Rangdo, Hiya (II), Niapin | 1039-1123 | 6 | Hill |
|  | Koloriang | Tayang, Buyang | 1053-1265 | 3 | Hill |
|  | Sarli | Sarli | 1425 | 1 | Hill |

^a^Approximate elevation using a handheld GPS unit at the village. The altitude of the rice fields will be higher, as those were located on the hill slopes

^b^Number of hill rice landraces collected from each village and as informed by the villagers

n/a, not available
