# Supplementary material for: Genetic diversity and structure in hill rice (Oryza sativa L.) landraces from the North-Eastern Himalayas of India
Source: BMC Genet. 2016 Jul 13;17:107. doi: 10.1186/s12863-016-0414-1 (PMC4944464; doi:10.1186/s12863-016-0414-1)
Supplement: Additional file 3: — Names, accession number, classification and genetic structure of 64 hill rice landraces and control varieties used in SSR fingerprinting. (DOC 135 kb) [file 12863_2016_414_MOESM3_ESM.doc]

**Additional file 3:** Names, accession number, classification and genetic structure of 64 hill rice landraces and control varieties used in SSR fingerprinting

| **Sample No.** | **Cultivar name** | **Accession No./Code** | **Collection site/ seed source** | **Classification** | **Farmers’ grouping** | **NJ cluster** | **Structure group^a^** |  |  |
| --- | --- | --- | --- | --- | --- | --- | --- | --- | --- |
|  |  |  |  |  |  |  | ***K ­*= 2** | ***K* = 3** | ***K* = 4** |
|  | **HILL RICE (n= 64)** |  |  |  |  |  |  |  |  |
| 1 | Pucho | IC0596595 | EK | Hill rice | *umte* | 4 | 1 | 3 | 4 |
| 2 | Umte | IC0610245 | EK | Hill rice | *umte* | 4 | admixture_1 | admixture_3 | admixture_3 |
| 3 | Naga Um | IC0596596 | EK | Hill rice | *umte* | 4 | 2 | 3 | 4 |
| 4 | Damchak | IC0610246 | EK | Hill rice | *tening* | 4 | 1 | admixture_1 | 3 |
| 5 | Kilung | IC0596597 | EK | Hill rice | *tening* | 4 | 2 | 3 | 4 |
| 6 | Adi | IC0610247 | EK | Hill rice | *tening* | 4 | 2 | 3 | 4 |
| 7 | Damchak | IC0596598 | EK | Hill rice | *tening* | 4 | 2 | 3 | 4 |
| 8 | Kilung | MR9 | EK | Hill rice | *tening* | 4 | 2 | 3 | 4 |
| 9 | Radang | IC0596599 | EK | Hill rice | *umte* | 4 | 2 | 3 | admixture_4 |
| 10 | Nishin | IC0596600 | EK | Hill rice | *umte* | 2 | 2 | admixture_3 | admixture_4 |
| 11 | Rimi | IC0596601 | EK | Hill rice | *tening* | 4 | admixture_2 | 3 | admixture_4 |
| 12 | Miri | IC0596602 | EK | Hill rice | *tening* | 4 | admixture_1 | admixture_1 | admixture_4 |
| 13 | Radang | MR15 | EK | Hill rice | *umte* | 3 | admixture_1 | admixture_3 | 3 |
| 14 | Ningpung | IC0610248 | EK | Hill rice | *umte* | 4 | admixture_2 | 3 | 4 |
| 15 | Radang | IC0610249 | EK | Hill rice | *umte* | 4 | admixture_1 | 3 | admixture_4 |
| 16 | Paun | IC0610250 | EK | Hill rice | *umte* | 4 | admixture_2 | 3 | 4 |
| 17 | Bibolilo | IC0610251 | EK | Hill rice | *umte* | 4 | 1 | 3 | 3 |
| 18 | Tezek | MR20 | EK | Hill rice | *tening* | 3 | 2 | 3 | 3 |
| 19 | Rungpchi | IC0610252 | EK | Hill rice | *umte* | 4 | admixture_1 | 3 | admixture_3 |
| 20 | Saap | IC0596603 | EK | Hill rice | *tening* | 4 | 2 | 3 | 4 |
| 21 | Tazek | IC0610253 | EK | Hill rice | *tening* | 4 | admixture_2 | 3 | admixture_4 |
| 22 | Bibolilo | MR24 | EK | Hill rice | *umte* | 3 | 2 | 3 | 3 |
| 23 | Itanagar dhan | IC0610254 | EK | Hill rice | *umte* | 4 | admixture_1 | admixture_3 | admixture_2 |
| 24 | Karta Pachung | MR27 | EK | Hill rice | *tening* | 2 | 2 | admixture_3 | admixture_4 |
| 25 | Nepali dhan | MR28 | EK | Hill rice | *umte* | 2 | 2 | admixture_3 | admixture_4 |
| 26 | Naga Dhan | MR29 | EK | Hill rice | *umte* | 1 | admixture_1 | admixture_1 | 3 |
| 27 | Karta Dhan | IC0596604 | EK | Hill rice | *tening* | 4 | admixture_2 | admixture_3 | admixture_4 |
| 28 | Ningte | MR31 | EK | Hill rice | *tening* | 2 | 2 | 2 | admixture_1 |
| 29 | Ningche | MR32 | EK | Hill rice | *tening* | 4 | 2 | 3 | 4 |
| 30 | Bumbe | IC0610255 | EK | Hill rice | *umte* | 4 | 2 | admixture_2 | admixture_1 |
| 31 | Rimi | MR34 | EK | Hill rice | *tening* | 4 | 2 | 2 | 1 |
| 32 | Jijiko | MR35 | EK | Hill rice | *umte* | 4 | admixture_2 | admixture_3 | admixture_4 |
| 33 | Rimi | IC0596605 | EK | Hill rice | *tening* | 4 | 2 | 2 | 1 |
| 34 | Farmers’ sel. | MR37 | EK | Hill rice | *umte* | 4 | 2 | 2 | 1 |
| 35 | Mapung | MR38 | PP | Hill rice | *tening* | 4 | 2 | 2 | 1 |
| 36 | Kabang | MR39 | PP | Hill rice | *tening* | 4 | 2 | 3 | 4 |
| 37 | Legmin | IC0610256 | PP | Hill rice | *tening* | 4 | 2 | admixture_3 | 4 |
| 38 | Naar | IC0610257 | PP | Hill rice | *tening* | 4 | 2 | 2 | 1 |
| 39 | Manipuri dhan | MR42 | PP | Hill rice | *tening* | 4 | 2 | 2 | 1 |
| 40 | Langma | MR43 | PP | Hill rice | *tening* | 4 | 2 | 2 | 1 |
| 41 | Serom | MR44 | PP | Hill rice | *tening* | 4 | 2 | 2 | 1 |
| 42 | Sarpung | MR45 | PP | Hill rice | *tening* | 4 | 2 | admixture_3 | admixture_4 |
| 43 | Taba | IC0610258 | PP | Hill rice | *tening* | 4 | 2 | 2 | 1 |
| 44 | Maglang | MR47 | PP | Hill rice | *tening* | 4 | 2 | 2 | 1 |
| 45 | Mopu | MR48 | PP | Hill rice | *tening* | 4 | 2 | 3 | 4 |
| 46 | Langme | IC0596606 | PP | Hill rice | *umte* | 4 | 2 | admixture_3 | 4 |
| 47 | Langme | IC0610259 | PP | Hill rice | *umte* | 4 | 2 | 2 | 1 |
| 48 | Jamek | MR51 | KK | Hill rice | *tening* | 4 | 2 | 2 | 1 |
| 49 | Umbo | IC0610260 | KK | Hill rice | *umte* | 4 | admixture_2 | 3 | admixture_4 |
| 50 | Umleng | IC0610261 | KK | Hill rice | *tening* | 4 | 1 | 1 | 2 |
| 51 | Charmui | IC0610262 | KK | Hill rice | *tening* | 4 | 1 | 1 | 2 |
| 52 | Taba dugu | MR55 | KK | Hill rice | *tening* | 4 | 2 | admixture_3 | admixture_4 |
| 53 | Umleng | IC0610263 | KK | Hill rice | *umte* | 4 | 1 | 1 | 2 |
| 54 | Phasi | MR57 | KK | Hill rice | *umte* | 4 | 2 | 3 | 4 |
| 55 | Tesang | IC0610264 | KK | Hill rice | *umte* | 4 | 1 | 1 | 2 |
| 56 | Tsabu | IC0610265 | KK | Hill rice | *umte* | 1 | 1 | 1 | 3 |
| 57 | Kilung | IC0596607 | KK | Hill rice | *tening* | 4 | 2 | admixture_3 | admixture_4 |
| 58 | Bamak | IC0610266 | KK | Hill rice | *tening* | 1 | 1 | admixture_1 | 3 |
| 59 | Kilung | MR62 | KK | Hill rice | *tening* | 4 | 2 | 3 | 4 |
| 60 | Umpa | IC0610267 | KK | Hill rice | *umte* | 1 | 1 | 1 | 3 |
| 61 | Umpa | IC0596608 | KK | Hill rice | *umte* | 4 | 2 | admixture_2 | admixture_1 |
| 62 | Sarpung | MR65 | KK | Hill rice | *tening* | 4 | 2 | 3 | 4 |
| 63 | Jagam | MR66 | KK | Hill rice | *tening* | 4 | 2 | 3 | 4 |
| 64 | Lungbo | IC0610268 | KK | Hill rice | *tening* | 1 | 1 | 1 | 3 |
|  | **CONTROLS (n = 15)** |  |  |  |  |  |  |  |  |
| 65 | IR8 | IC0078688 | NRRI | *indica* | - | 1 | 1 | 1 | 2 |
| 66 | IR36 | IC0075523 | NRRI | *indica* | - | 1 | 1 | 1 | 2 |
| 67 | Jaya | IC0074299 | NRRI | *indica* | - | 1 | 1 | 1 | 2 |
| 68 | Taichung Native 1 | IC0459827 | NRRI | *indica* | - | 1 | 1 | 1 | 2 |
| 69 | TKM9 | IC0070839 | NRRI | *indica* | - | 1 | admixture_1 | admixture_3 | admixture_3 |
| 70 | FR 13A | IC0454299 | NRRI | *aus* | - | 1 | 1 | 1 | 3 |
| 71 | Kalamkati | IC0450351 | NRRI | *aus* | - | 1 | 1 | 1 | 3 |
| 72 | Basmati370 | IC0210758 | NRRI | Basmati (*aromatic*) | - | 2 | 1 | admixture_1 | 3 |
| 73 | Pakistani Basmati | IC0279697 | NRRI | Basmati (*aromatic*) | - | 2 | admixture_1 | admixture_3 | admixture_3 |
| 74 | Pusa Basmati | IC0443776 | NRRI | Basmati (*aromatic*) | - | 2 | 1 | 1 | admixture_3 |
| 75 | Badshabhog | IC0123551 | NRRI | Small-grain aromatic | - | 2 | 1 | admixture_1 | 3 |
| 76 | Kataribhog | IC0450334 | NRRI | Small-grain aromatic | - | 2 | 1 | admixture_1 | 3 |
| 77 | Mohanbhog | IC012478 | NRRI | Small-grain aromatic | - | 2 | 1 | 3 | 3 |
| 78 | Azucena | EC0391385 | NRRI | *tropical japonica* | - | 4 | admixture_1 | 3 | admixture_4 |
| 79 | Dular | IC0519994 | NRRI | *japonica* admixture | - | 3 | admixture_2 | admixture_2 | admixture_3 |

EK, East Kameng district

PP, Papum Pare district

KK, Kurung Kumey district

NRRI, ICAR-National Rice Research Institute, Cuttack, Odisha

^a^Accessions with <80% inferred ancestry from any one group were designated as admixture
