# Supplementary material for: Genetic diversity and structure in hill rice (Oryza sativa L.) landraces from the North-Eastern Himalayas of India
Source: BMC Genet. 2016 Jul 13;17:107. doi: 10.1186/s12863-016-0414-1 (PMC4944464; doi:10.1186/s12863-016-0414-1)
Supplement: Additional file 4: — Details and summary statistics of 35 SSR markers used in the study. (DOC 73 kb) [file 12863_2016_414_MOESM4_ESM.doc]

**Additional file 4:** Details and summary statistics of 35 SSR markers used in the study

| **Marker** | **Chr** | **Size range (bp)** | **n** | **MAF** | ***H_e_*** | ***H_o_*** | ***R_s_*** | **PIC** |
| --- | --- | --- | --- | --- | --- | --- | --- | --- |
| RM431 | 1 | 240-258 | 11 | 0.27 | 0.85 | 0.00 | 8.62 | 0.84 |
| RM1 | 1 | 76-119 | 14 | 0.47 | 0.72 | 0.04 | 7.63 | 0.69 |
| RM259 | 1 | 148-184 | 23 | 0.10 | 0.94 | 0.06 | 14.55 | 0.93 |
| RM495^*^ | 1 | 147-159 | 3 | 0.64 | 0.52 | 0.09 | 2.98 | 0.46 |
| RM5 | 1 | 100-126 | 9 | 0.34 | 0.80 | 0.10 | 7.51 | 0.78 |
| RM154 | 2 | 165-199 | 12 | 0.23 | 0.85 | 0.08 | 8.42 | 0.83 |
| RM208^*^ | 2 | 164-178 | 8 | 0.61 | 0.60 | 0.06 | 6.07 | 0.58 |
| RM489 | 3 | 235-271 | 5 | 0.42 | 0.72 | 0.00 | 4.30 | 0.67 |
| RM55 | 3 | 217-236 | 6 | 0.76 | 0.41 | 0.00 | 4.55 | 0.39 |
| RM338 | 3 | 179, 182 | 2 | 0.51 | 0.50 | 0.00 | 2.00 | 0.37 |
| RM514 | 3 | 237-271 | 7 | 0.39 | 0.73 | 0.06 | 5.62 | 0.69 |
| RM124 | 4 | 265-271 | 3 | 0.44 | 0.65 | 0.00 | 3.00 | 0.58 |
| RM307 | 4 | 120-177 | 11 | 0.25 | 0.82 | 0.04 | 7.08 | 0.80 |
| RM507 | 5 | 254-262 | 3 | 0.75 | 0.41 | 0.00 | 2.92 | 0.37 |
| RM413 | 5 | 69-111 | 9 | 0.61 | 0.59 | 0.10 | 5.40 | 0.57 |
| RM178 | 5 | 114-121 | 4 | 0.53 | 0.60 | 0.00 | 3.29 | 0.53 |
| RM334 | 5 | 143-243 | 14 | 0.35 | 0.82 | 0.09 | 8.86 | 0.80 |
| RM133 | 6 | 228-235 | 5 | 0.59 | 0.53 | 0.00 | 3.27 | 0.44 |
| RM510 | 6 | 109-127 | 7 | 0.32 | 0.78 | 0.00 | 5.56 | 0.75 |
| RM162 | 6 | 201-244 | 12 | 0.18 | 0.86 | 0.05 | 8.22 | 0.85 |
| RM125 | 7 | 120-138 | 6 | 0.62 | 0.58 | 0.01 | 5.11 | 0.55 |
| RM11 | 7 | 119-143 | 10 | 0.19 | 0.87 | 0.06 | 8.29 | 0.85 |
| RM118 | 7 | 157-162 | 4 | 0.53 | 0.59 | 0.00 | 3.28 | 0.51 |
| RM152 | 8 | 132-155 | 5 | 0.53 | 0.66 | 0.06 | 4.85 | 0.62 |
| RM25 | 8 | 126-155 | 12 | 0.23 | 0.87 | 0.01 | 8.94 | 0.85 |
| RM284 | 8 | 140-150 | 6 | 0.56 | 0.63 | 0.00 | 5.05 | 0.59 |
| RM433 | 8 | 219-235 | 8 | 0.45 | 0.72 | 0.00 | 6.18 | 0.69 |
| RM316 | 9 | 187-296 | 13 | 0.31 | 0.84 | 0.10 | 9.46 | 0.83 |
| RM105 | 9 | 126-136 | 5 | 0.49 | 0.68 | 0.00 | 4.71 | 0.64 |
| RM271 | 10 | 86-116 | 11 | 0.58 | 0.63 | 0.27 | 6.08 | 0.60 |
| RM171 | 10 | 324-348 | 5 | 0.38 | 0.71 | 0.03 | 4.20 | 0.65 |
| RM552 | 11 | 173-228 | 20 | 0.25 | 0.86 | 0.27 | 10.07 | 0.84 |
| RM536 | 11 | 212-238 | 11 | 0.23 | 0.84 | 0.00 | 7.62 | 0.82 |
| RM19 | 12 | 214-248 | 8 | 0.67 | 0.52 | 0.21 | 4.88 | 0.49 |
| RM277 | 12 | 114-122 | 5 | 0.58 | 0.52 | 0.00 | 2.91 | 0.42 |
| **Mean** |  | **-** | **8.49** | **0.44** | **0.69** | **0.05** | **6.04** | **0.65** |

Chr, Rice chromosome

n, Number of alleles

MAF*,* Major allele frequency (the alleles with the highest frequency)

*H_e_*, expected heterozygosity

*H_o_*, observed heterozygosity

*R_S_*, average allelic richness

PIC, Polymorphic information content

^*^Markers excluded from the combined analysis of 64 hill rices and 234 global rices
