# Supplementary material for: Genetic diversity and structure in hill rice (Oryza sativa L.) landraces from the North-Eastern Himalayas of India
Source: BMC Genet. 2016 Jul 13;17:107. doi: 10.1186/s12863-016-0414-1 (PMC4944464; doi:10.1186/s12863-016-0414-1)
Supplement: Additional file 6: — Determination of the best K value(s) based on mean LnP(D) over five runs for each K value, and rate of change in the log probability of data between successive K values (∆K). (PDF 1363 kb) [file 12863_2016_414_MOESM6_ESM.pdf]

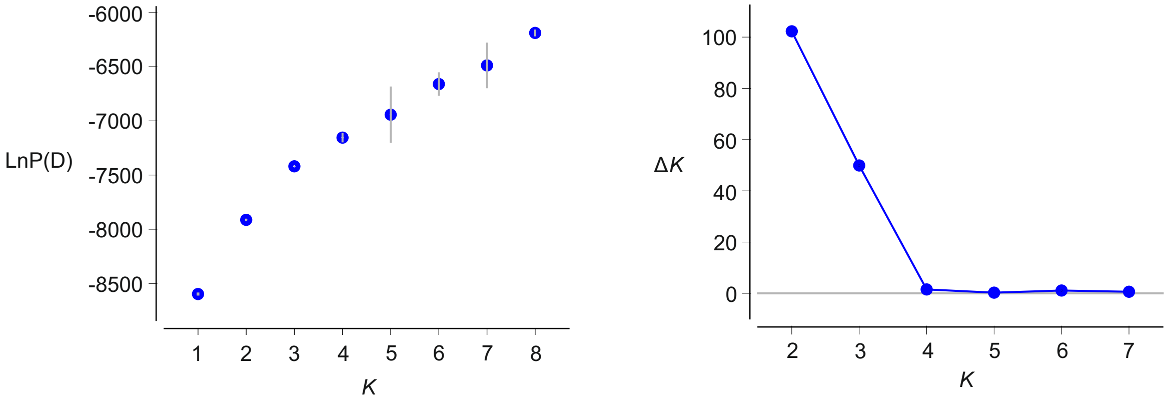

Additional file 6: Determination of the best *K* value(s) based on mean LnP(D) over five runs for each *K* value, and rate of change in the log probability of data between successive *K* values (ΔK)
