# Supplementary material for: Genetic diversity and structure in hill rice (Oryza sativa L.) landraces from the North-Eastern Himalayas of India
Source: BMC Genet. 2016 Jul 13;17:107. doi: 10.1186/s12863-016-0414-1 (PMC4944464; doi:10.1186/s12863-016-0414-1)
Supplement: Additional file 7: — Analysis of SSR molecular variance. (DOC 32 kb) [file 12863_2016_414_MOESM7_ESM.doc]

**Additional file 7:** Analysis of SSR molecular variance

| **Population** | **df** | **Percentage of variation** |  | ***p* value** |
| --- | --- | --- | --- | --- |
|  |  | **Among groups** | **Within groups** |  |
| Districts | 2 | 7.3 | 92.7 | 0.001 |
| Farmers’ classified groups | 1 | 0.8 | 99.2 | 0.089 |
| Structure subpopulations | 3 | 14.3 | 85.7 | 0.001 |
| Hill rice and controls | 1 | 9.1 | 90.9 | 0.001 |
