# Supplementary material for: Genetic diversity and structure in hill rice (Oryza sativa L.) landraces from the North-Eastern Himalayas of India
Source: BMC Genet. 2016 Jul 13;17:107. doi: 10.1186/s12863-016-0414-1 (PMC4944464; doi:10.1186/s12863-016-0414-1)
Supplement: Additional file 8: — Pair-wise F st values for different sub-populations. (DOC 58 kb) [file 12863_2016_414_MOESM8_ESM.doc]

**Additional file 8:** Pair-wise *F_st_* values for different subpopulations

|  | PP | KK | *Umte* | *Tening* | Control | StrGr1 | StrGr2 | StrGr3 | StrGr4 | Hill | *indica* | *aus* | *aromatic* | *TRJ* | *TEJ* | admixture |
| --- | --- | --- | --- | --- | --- | --- | --- | --- | --- | --- | --- | --- | --- | --- | --- | --- |
| EK | 0.083 | 0.043 | - | - | 0.091 | - | - | - | - | - | - | - | - | - | - | - |
| PP |  | 0.126 | - | - | 0.177 | - | - | - | - | - | - | - | - | - | - | - |
| KK |  |  | - | - | 0.084 | - | - | - | - | - | - | - | - | - | - | - |
| *Umte* |  |  |  | 0.008 | 0.072 | - | - | - | - | - | - | - | - | - | - | - |
| *Tening* |  |  |  |  | 0.104 | - | - | - | - | - | - | - | - | - | - | - |
| Control |  |  |  |  |  | - | - | - | - | - | - | - | - | - | - | - |
| StrGr1 |  |  |  |  |  |  | 0.290 | 0.155 | 0.129 | - | - | - | - | - | - | - |
| StrGr2 |  |  |  |  |  |  |  | 0.120 | 0.210 | - | - | - | - | - | - | - |
| StrGr3 |  |  |  |  |  |  |  |  | 0.086 | - | - | - | - | - | - | - |
| StrGr4 |  |  |  |  |  |  |  |  |  | - | - | - | - | - | - | - |
| Hill |  |  |  |  |  |  |  |  |  |  | 0.231 | 0.183 | 0.144 | 0.189 | 0.235 | 0.095 |
| *indica* |  |  |  |  |  |  |  |  |  |  |  | 0.209 | 0.348 | 0.403 | 0.462 | 0.296 |
| *aus* |  |  |  |  |  |  |  |  |  |  |  |  | 0.269 | 0.398 | 0.472 | 0.234 |
| *aromatic* |  |  |  |  |  |  |  |  |  |  |  |  |  | 0.279 | 0.417 | 0.116 |
| *TRJ* |  |  |  |  |  |  |  |  |  |  |  |  |  |  | 0.300 | 0.140 |
| *TEJ* |  |  |  |  |  |  |  |  |  |  |  |  |  |  |  | 0.198 |

Geographic regions: EK, East Kameng; PP, Papum Pare; KK, Kurung Kumey

Farmers’ classified groups in 64 hill rice: *Umte* and *Tening*

Control, 15 control varieties used in the study

STRUCTURE subpopulations (*K* = 4) of 79 hill and control varieties: StrGr1-4

Subpopulations in combined analysis: Hill, 64 hill rice accessions and subgroups in reference set including *indica*, *aus*, *aromatic*, *tropical japonica* (*TRJ*), *temperate japonica* (*TEJ*) and admixture
