# Supplementary material for: Genetic diversity and structure in hill rice (Oryza sativa L.) landraces from the North-Eastern Himalayas of India
Source: BMC Genet. 2016 Jul 13;17:107. doi: 10.1186/s12863-016-0414-1 (PMC4944464; doi:10.1186/s12863-016-0414-1)
Supplement: Additional file 9: — Phylogenetic reconstruction of 150 reference rice accessions (Garris et al., 2005) based on 33 SSR markers data. (PDF 1358 kb) [file 12863_2016_414_MOESM9_ESM.pdf]

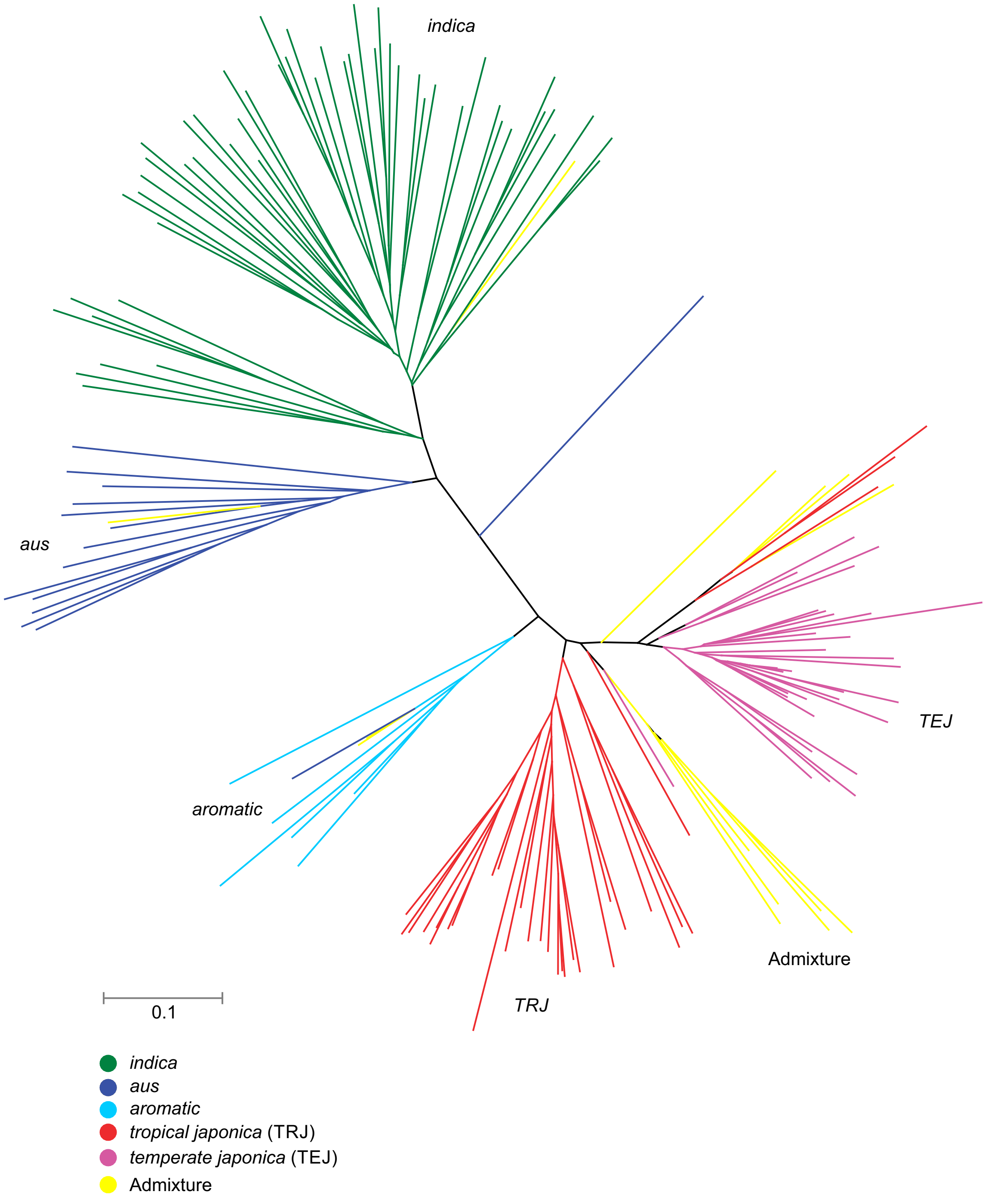

Additional file 9: Phylogenetic reconstruction of 150 reference rice accessions (Garris et al., 2005) based on 33 SSR markers data
